# Mining Virulence Genes Using Metagenomics

**DOI:** 10.1371/journal.pone.0024975

**Published:** 2011-10-19

**Authors:** Pedro Belda-Ferre, Raúl Cabrera-Rubio, Andrés Moya, Alex Mira

**Affiliations:** 1 Joint Unit of Research in Genomics and Health, Centre for Public Health Research-Cavanilles Institute for Biodiversity and Evolutionary Biology, University of Valencia, Valencia, Spain; 2 Centro de Investigación Biomédica en Red especializado en Epidemiología y Salud Pública, Madrid, Spain; Argonne National Laboratory, United States of America

## Abstract

When a bacterial genome is compared to the metagenome of an environment it inhabits, most genes recruit at high sequence identity. In free-living bacteria (for instance marine bacteria compared against the ocean metagenome) certain genomic regions are totally absent in recruitment plots, representing therefore genes unique to individual bacterial isolates. We show that these Metagenomic Islands (MIs) are also visible in bacteria living in human hosts when their genomes are compared to sequences from the human microbiome, despite the compartmentalized structure of human-related environments such as the gut. From an applied point of view, MIs of human pathogens (e.g. those identified in enterohaemorragic *Escherichia coli* against the gut metagenome or in pathogenic *Neisseria meningitidis* against the oral metagenome) include virulence genes that appear to be absent in related strains or species present in the microbiome of healthy individuals. We propose that this strategy (i.e. recruitment analysis of pathogenic bacteria against the metagenome of healthy subjects) can be used to detect pathogenicity regions in species where the genes involved in virulence are poorly characterized. Using this approach, we detect well-known pathogenicity islands and identify new potential virulence genes in several human pathogens.

## Introduction

Identifying virulence genes experimentally is one of the cornerstones of bacterial pathogenesis research. Experimental approaches typically include cloning of genes potentially involved in pathogenesis into a laboratory strain, transposon mutagenesis to generate a collection of mutants, or detection of genes essential for survival in the host by *in vivo* expression technology [1]. The completion of bacterial genomes now allows to directly detecting genes that could be involved in pathogenicity: when both pathogenic and commensal strains of the same species are sequenced, the genes unique to the pathogen can easily be located [2]. The selection of potential candidate genes is more refined as the number of non-pathogenic strains for comparison increases. Thus, an ideal comparison would be provided by a pathogenic strain and a whole population of related, avirulent strains inhabiting the human body of a healthy individual. The advent of metagenomics and its application to the study of the human microbiome [3], [4] now provides a unique opportunity to perform these comparisons, as the total gene pool from whole microbial populations can be compared against the genome of individual pathogenic strains.

A fast way to make these comparisons is achieved by metagenomic recruitments [5]. Individual metagenomic reads that give a hit over a certain identity threshold against a reference bacterial genome are “recruited” to plot a graph which will vary in density depending on the abundance of that organism in the sample. Interestingly, it has frequently been found that recruitments of marine bacteria against all marine metagenomes available identified several “islands” of extremely limited or absent coverage, even for species which were dominant in the sample [6]. These “Metagenomic Islands” have also been found in other free-living environments [7] and represent segments of the genome which are highly variable or specific to the reference strain. Assuming that virulent strains are absent from healthy individuals, metagenomic recruitments of pathogenic strains of bacteria whose commensal counterparts are typically found in the human microbiome should reveal MIs at the regions where virulence genes are located. To test this possibility we have compared the genomes of several human pathogens against available gut metagenomes and against several oral metagenomes obtained by ourselves and other groups through direct pyrosequencing from oral cavity samples.

## Results

### Human-associated bacteria display Metagenomic Islands (MI)

**Figure 1:**
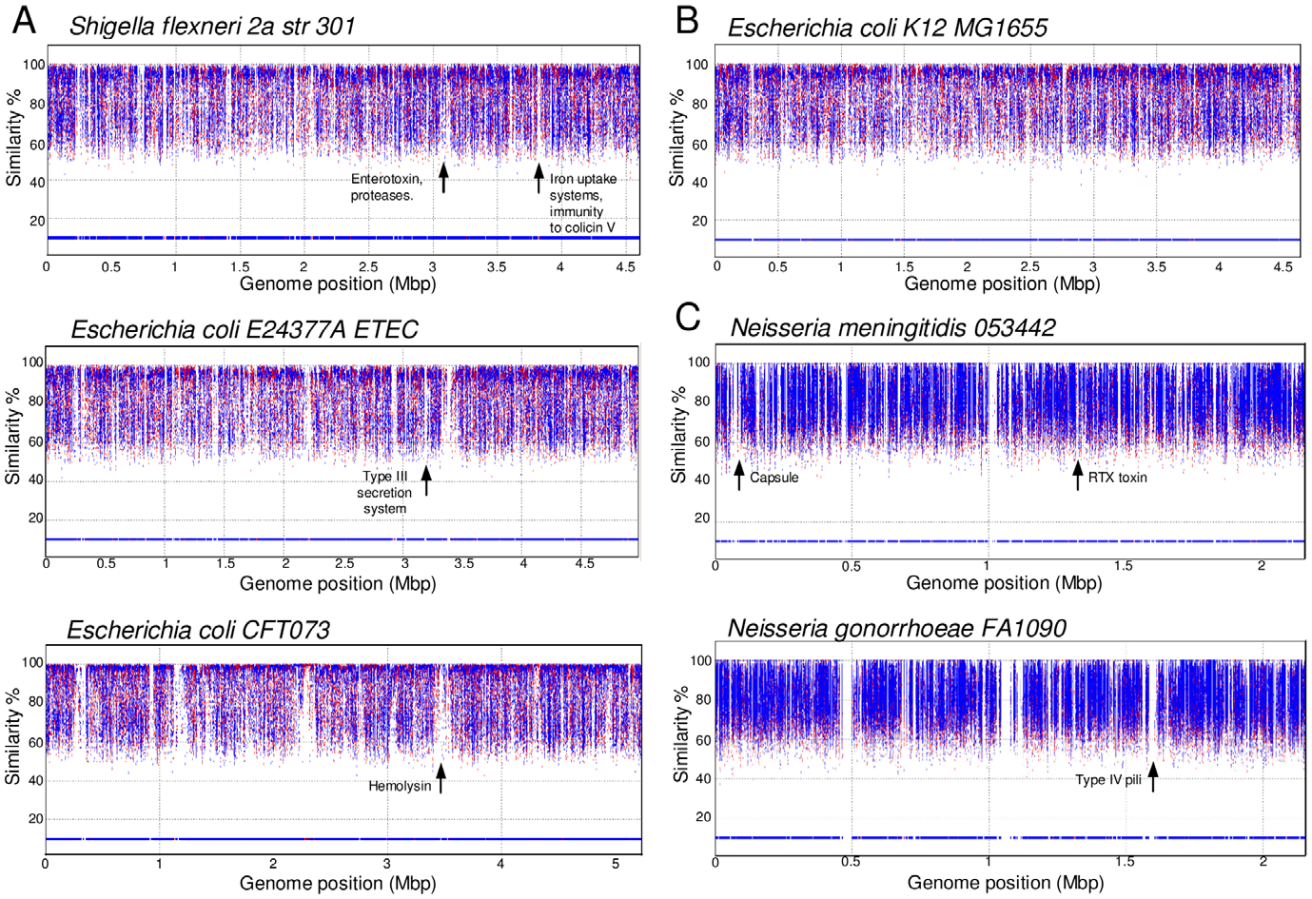
Comparing healthy microbiomes against bacterial genomes. Metagenomic recruitment of (A) the gut metagenome against the three enteric human pathogens *S. flexneri*. *E. coli* ETEC and *E. coli* CFT073; (B) the gut metagenome against the avirulent *E. coli* K12 laboratory strain; and (C) the dental plaque metagenome against twoe pathogenic neisserial species. Some relevant pathogenicity islands are indicated (for a full list of MIs gene content see Table S1). The few islands detected in the commensal *E. coli* K12 strain correspond to mobile genetic elements, mainly phage genes, as well as a few outer membrane genes.

Similarly to free-living bacteria, when metagenomic recruitments are made between the genomes of gut-associated bacteria against the human gut metagenome, regions with low or absent recruitment are clearly visible (Figure 1A). This shows that gut inhabitants also have genomic regions that appear to be unique to individual strains. In free-living habitats like aquatic environments, intraspecific genomic diversification has been proposed as a strategy to exploit different microniches [8], and this would partly account for differences in gene content among strains from the same species. In the gut and other host-related environments, the confinement of bacteria to a given host individual, with limited microbial exchange through the faecal-oral route, imposes a compartmentalized structure to these niches, which may contribute to the observed differences in genomic content between strains. However, the appearance of MIs could also be showing genes specific to virulent strains because recruitment plots for commensal bacteria displayed a higher coverage along the genome and a limited presence of MIs (Fig. 1B), which were limited to mobile genetic elements, mainly phage genes (48.8% of the total) and several outer membrane proteins (14.6% of the total). Thus, in order to determine whether regions of absent recruitment identify genes involved in pathogenicity, we performed a systematic description of gene content in MIs from human pathogens for which pathogenicity islands and virulence genes are well characterized.

### MIs identify virulence genes

As shown in Figure 1A, recruitments of human pathogens against the gut metagenome of healthy individuals show MIs which correspond to virulence genes. The gene content of all MIs identified in pathogenic *Shigella* and *Escherichia* strains against the gut metagenome (Table S1) also reveals, as expected, the presence of mobile elements like IS elements and phage genes. In fact, prophages appear to be quite unique to individual strains and represent an important portion of MIs. This may reflect viral infection specificity for individual strains and also high divergence rates for genes which are among the fastest evolving in microbial genomes [6]. But apart from mobile elements, a large proportion of MIs was formed by genes shown experimentally to be involved in pathogenesis and other well-known virulence factors. These include fimbrial proteins, toxins, type I, II and III secretion systems, cell invasion proteins and various antigens, among others (Table S1).

A similar pattern was found when pathogenic Neisserial and Streptococcal species were compared against the oral metagenome of healthy individuals (Figure 1C). The oral microbiome is known to be rich in commensal Neisserial and Streptococcal species [9] and therefore the MIs, apart from containing mobile genetic elements, included many genes involved in pathogenesis such as well-characterized toxins, antigens, hemolysins and adhesins (Table S1). In addition to experimentally demonstrated virulence factors, the islands include many ORFs of unknown function, some of which could also be involved in pathogenesis and should therefore be characterized. An example is given by a 5.7 Kb MI in *Streptococcus pneumoniae* R6, where only hypothetical proteins are annotated (Table S1). Refined sequence similarity searches show that the second half of the island contains genes with homology to the *fmt*A protein family, which modulates antibiotic resistance in *Staphylococcus aureus* [13] and adds to other antibiotic resistance genes found in other islands.

When known pathogenicity islands from seven well characterized pathogens were compared to the MIs identified in the present study, most virulence genes were detected (Table 1). Some of the genes which have been shown to play a role in virulence are not detected in MIs because they are involved in several vital cellular functions other than pathogenicity and therefore they are also found in non-virulent strains. These include iron uptake systems or genes involved in adherence to the host. However, most genes directly participating in virulence like toxins, immune evasion systems or proteins involved in cell invasion were readily identified.

**Table 1:** Detection of virulence genes in Metagenomic Islands (MIs).

SPECIES	FUNCTION	VIRULENCE GENES	NUMBER OF GENES IN MI
*Neisseria meningitidis FAM18*	Adherence	27	13
	Immune evasion	11	11
	Invasion	6	5
	Iron uptake systems	14	7
	IgA protease	1	1
	Toxin	2	2
*Neisseria gonorrhoeae FA 1090*	Adherence	24	12
	Immune evasion	0	
	Invasion	13	12
	Iron uptake systems	12	6
	IgA protease	1	1
	Toxin	1	0
*Shigella flexneri 2a str. 301 chromosome*	Host immune evasion	3	3
	Iron uptake systems	20	6
	Protease	2	1
	Secretion system	7	7
	Toxin	2	2
*Shigella flexneri 2a str. 301 plasmid*	Protease	2	1
	Secretion system	52	51
	Others	4	2
*Escherichia coli CFT073*	Adherence	45	8
	Autotransporter	4	2
	Iron uptake systems	33	7
	Toxins	4	2
*Escherichia coli O157:H7 str. Sakai*	Adherence	17	2
	Autotransporter	1	0
	Iron uptake systems	7	0
	LEE encoded TTSS effectors	6	6
	Non-LEE encoded TTSS effectors	5	5
	Secretion system	34	32
	Toxins	4	4
*E. coli O157:H7 str. Sakai plasmid O157*	Adherence	1	1
	Autotransporter	1	1
	Toxin	4	3

Experimentally characterized virulence genes were obtained from the Virulence Factors Database [14].

**Figure 2:**
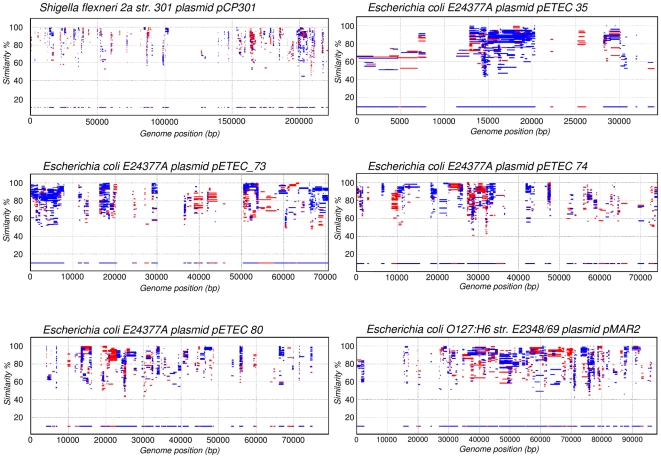
Detecting virulence plasmids by metagenomics. Protein recruitment plots obtained by comparing the healthy gut metagenome against plasmids of pathogenic *Shigella flexneri* and *E. coli* strains. The islands identify known virulence genes whereas genes involved in plasmid housekeeping functions display high recruitment (see Table S2).

Given that many virulence genes are coded in extrachromosomal elements, the same approach was followed for well-characterized bacterial plasmids of enteric bacteria. Despite the promiscuous nature of many extrachromosomal replicons, most plasmids genes from pathogenic strains of *E. coli* showed an intense coverage (Figure 2), showing that these are frequent among natural populations of commensal enteric bacteria. However, clear islands were also identified. Examination of gene content in plasmids' MIs indicated that virulence genes were again absent from the recruitments (Table S2), whereas genes involved in replication, conjugation and other basic plasmid functions were well represented in the gut metagenome. Thus, metagenomic recruitments can prove useful to detect virulence plasmids and to determine which regions from an uncharacterized plasmid may be involved in pathogenicity.

### Detection of new virulence genes in Streptococci

We have applied the proposed method in two streptococcal species which vary in their degree of study and pathogenicity. *S. sanguinis* is a normal, commensal inhabitant of the human mouth but after surgeries it can enter the bloodstream and cause endocarditis. When doing recruitment plots of its genome against the oral metagenome of healthy individuals, several MIs were identified (Table S1). Two of them, containing ORFs coding for a platelet binding glycoprotein and adhesion proteins had already been described as virulence factors [10], showing that the method detects known virulence genes. In addition, an 8.9 Kb metagenomic island contains a hemolysin gene which could have a role in red-blood cell lysis and another 6 Kb island includes a gene with high similarity to a precursor of surface antigens (see Table S1). *S. pneumoniae* is a major human pathogen, and different strains are involved in many types of infection ranging from pneumonia to otitis media, meningitis, endocarditis or bacteremia. We have compared the genome of strain R6 against the healthy oral metagenome and have also found new genes potentially involved in virulence (Table S1). These include several *lic* genes, which have been shown to be involved in adherence and nasopharyngeal colonization in animal models [11], and an exoribonuclease from the VacB family, which has been shown to be involved in virulence in enteric bacteria [12]. Genes with significant homology to an immunoglobulin protease, a type IV prepilin peptidase and several cell wall anchor proteins were also within different islands. In several occasions, the islands were located at positions where no genes had been annotated (Table S1). BlastX searches within these regions, however indicated homology to several proteins, like ORFs with repetitive domains and significant homology to hydrolases, fatty acid metabolism proteins and LPXTG-motif cell wall anchor domains (position 556,709 in the R6 genome), suggesting that virulence factors could be present in these islands where functional genes could have passed unnoticed in annotation procedures. Thus, we propose the above genes as potentially involved in pathogenicity and suggest that similar analyses can be helpful in other human or animal pathogens.

### Conclusion

Although virulence is a complex trait determined by a large amount of genes and subject to intricate regulation (4% of *Salmonella typhimurium* has been shown to be involved in pathogenesis [14]) the simple procedure described here readily pinpoints most virulence genes unique to pathogenic strains. The procedure can be applied to other non-human pathogens. For instance, the genomes of fish pathogens can be compared against the metagenomes of marine samples where pathogenic strains of the same species are expected to be at low frequencies, and the healthy rumen metagenome could be used for comparison against pathogens of domestic farm animals. The presence of close relatives of plant pathogens in soil may also proof useful to determine genes involved in plant infection by comparing their genomes against the soil metagenome. As shown here, the recruitment of genomes from pathogens against the metagenome of healthy individuals containing commensal strains of the same species may prove extremely useful to select genes potentially involved in virulence and can be specially fruitful in species for which genes involved in pathogenicity are poorly characterized. The method has the advantage of using already-available sequenced metagenomes, whose number is rapidly increasing with the advent of more efficient and inexpensive sequencing techniques. In the future, this approach can be taken one step further when the metagenomes of diseased individuals are also available: In those cases, it will be possible to check whether the potential virulence determinants which are absent in the metagenomes of healthy individuals appear to be present in individuals with diseases such as Crohn's or ulcerative colitis. Obviously, once the approach proposed in this manuscript has narrowed down the list of potential candidates, such pathogenic capability must be tested experimentally. We anticipate that the use of MIs may reduce the number of genes to be cloned or mutated, therefore facilitating the basic process of characterizing virulence factors.

## Methods

### Oral DNA samples

A 22 year old caucasian healthy female participated in the study after signing informed consent. Sampling procedure was approved by the Ethical Committee for Clinical Research from the Center for Public Health Research (CEIC-DGSP/CSISP). The subject had not received any antibiotic treatment in the previous 2 months, had never suffered from caries and had no symptoms of gingivitis or gum bleeding at the sampling time. The volunteer was asked not to brush her teeth 24 hours before sample collection and not to eat in the prior 2 hours. Supragingival dental plaque was taken from all surfaces of all teeth with sterile toothpicks and pooled into a single sample. DNA was extracted using the AquaPure DNA extraction kit (BIORAD) following the manufacturer instructions and stored at −20°C. DNA concentration was measured with NanoDrop (Thermo Scientific), giving a 497.17 ng/µl concentration and a 260/280 ratio of 1.81 before pyrosequencing.

### Gut metagenomes

The sequences of the gut metagenome used were retrieved from the NCBI ftp site (ftp://ftp.ncbi.nih.gov/genbank/wgs/), and were composed of 15 healthy individuals, two subjects from Gill et al. (2006) [3], and 13 subjects from Kurokawa et al. (2007) [4], together accounting for a total of 804 Mbp of high-quality reads obtained by shot-gun and subsequent Sanger sequencing.

### Oral metagenomes

Oral DNA samples were sequenced using the GS-FLX pyrosequencer with the Titanium chemistry at Macrogen Inc, South Korea. A total of 175,401 reads were obtained, with an average length of 448 bp, adding to a total of 78.6 Mbp. Artificially replicated sequences that systematically appear in 454 data [15] were removed from the dataset using the “454 Replicate Filter” (http://microbiomes.msu.edu/replicates/). For that purpose, sequences that clustered together by CD-HIT and had their first 10 positions exactly identical, were replaced by the longest sequence in each cluster. Those spurious replicates accounted for 4.31% from the total. Additionally, reads corresponding to contaminating human DNA were also removed from the dataset by Megablast [16] against the human genome, with a E-value threshold of 1e-10, giving a final set of filtered sequences of 167,793 reads. These filtered reads have been deposited in the metagenome database from MG-RAST, with Accession Number 4447098.3. In addition to the metagenome we obtained, 113,312 pyrosequences from Xie et al. (2010) [17], accounting for 45.12 Mbp (MG-RAST ID:4446622.3) of sequence from a human dental plaque sample, were also added to the oral dataset, which contained 120.1 Mbp of high-quality sequence.

### Reference genomes

The genomes of pathogenic bacteria used in this study were retrieved from the NCBI FTP site (ftp://ftp.ncbi.nih.gov/genomes/Bacteria/). The annotation of those genomes were also downloaded in the. ptt format, in order to search for the function of genes within metagenomic islands. A list of the pathogen reference strains used with their genomes' accession IDs is supplied in Table S3.

### Detection of Metagenomic Islands through Recruitment plots

The metagenomic reads were mapped against the sequenced reference genomes using the Nucmer and Promer v3.06 alignment algorithms, with the default parameters [18]. To visualize data, results were plotted using Mummerplot, adding the coverage option, which plots all matches in one dimension, so areas of no recruitment can be readily detected. Using the coordinates files, we considered Metagenomic Islands (MI) as those genomic regions spanning one or more genes which gave no significant hits when mapping against the metagenomic reads at the protein level. For each MI, the genes annotated in that region were identified from the corresponding ptt files.

### Virulence genes

Information about virulence factors was obtained from the Virulence Factors Database (http://www.mgc.ac.cn/VFs/main.htm) [10] and from the Pathogenicity Island Database (http://www.gem.re.kr/paidb/) [19]. All virulence genes from the selected genomes were searched for within the detected MIs, thus the proportion of virulence genes contained inside the MIs could be quantified.

### Comparisons against different strains, species or genera

An important aspect of metagenome recruitments is to know against which bacterial strains in the metagenome are we comparing the reference genome. It has been shown that the average nucleotide identity (ANI) between orthologous genes of different strains within the same species is on average above 94% [20]. In fact, a threshold of 95% has been proposed as a substitute for the classical DNA-DNA hibridization assays for taxonomical assignments of new species [21]. Values of ANI between 90–95% are typically found for homologous genes in genome comparisons between different species of the same genus [20], [22]–[24]. Other thresholds for higher taxonomic levels are more difficult to establish, although they have been estimated for several two-way comparisons. For instance, the ANI for orthologous genes between *Escherichia* and *Salmonella* appears to be around 80% [25]. Thus, when comparing the reference genomes against the gut and oral metagenomes, a frequency histogram was made with the nucleotide identity obtained for each sequence (Figure S1). When the mode of the histogram was above 94%, the metagenomic recruitment was considered to correspond to strains of the same species. This is the case for the recruitment of *E. coli* O157:H7 against the healthy gut metagenome (Fig. S1A), where this pathogen is probably compared against the gut population of *E. coli* commensal strains. When *Neisseria meningitidis* is compared against the healthy oral metagenome, the peak in the frequency histogram is located at 91% nucleotide identity (Fig. S1B), indicating that this species is absent in the metagenome and therefore the recruitment is done against other Neisserial species which are common commensal inhabitants from the dental plaque [26]. A third case is provided by the recruitment of the typhi serovar of *Salmonella enterica*, a species which is absent from the healthy human gut, providing a peak in the frequency histogram at 81% nucleotide identity (Fig. S1C). Thus, the plot in this case shows the recruitment of *S. typhi* genes against a different species, primarily against the *E. coli* gut population. Even in the latter case, MIs are readily seen. However, the islands identified will represent not only genes unique to that strain within *Salmonella enterica*, but also genes present in all *Salmonella* species but absent in *E. coli*, making the comparison less specific. The most informative recruitments are therefore those made between a pathogenic strain against the non-pathogenic strains from the same species.

## Supporting Information

Figure S1 **Nucleotide recruitment plots obtained from comparing the intestinal metagenome against the genome of** ***Escherichia coli O157:H7 Sakai str.*** *(* ***A*** *)* and *Salmonella enterica subsp. enterica serovar Typhi str. CT18* (***C***), and from the oral metagenome against *Neisseria meningitidis FAM18* (***B***). Graphs on the right show frequency histograms of the similarity values of the reads mapped to a given genome. The black line marks the 94% standard threshold for mean identity values for strains from the same species. Taking this line as a threshold, the recruitments are performed against bacteria from the same species as the reference genome (A), different species from the same genus (B) or against a different genus (C).
(TIF)

Table S1 **Full gene content of Metagenomic Islands detected by recruitment of selected pathogenic species of pathogenic bacteria against the gut and oral metagenomes.**
(PDF)

Table S2 **Full gene content of Metagenomic Islands detected by recruitment of selected virulence plasmids from enteric bacteria against the gut metagenome.**
(PDF)

Table S3 **List of strains analyzed in the manuscript and their corresponding NCBI accession numbers.**
(PDF)
