# Supplementary figures and images for: Mining Virulence Genes Using Metagenomics
Source: PLoS One. 2011 Oct 19;6(10):e24975. doi: 10.1371/journal.pone.0024975 (PMC3198465; doi:10.1371/journal.pone.0024975)

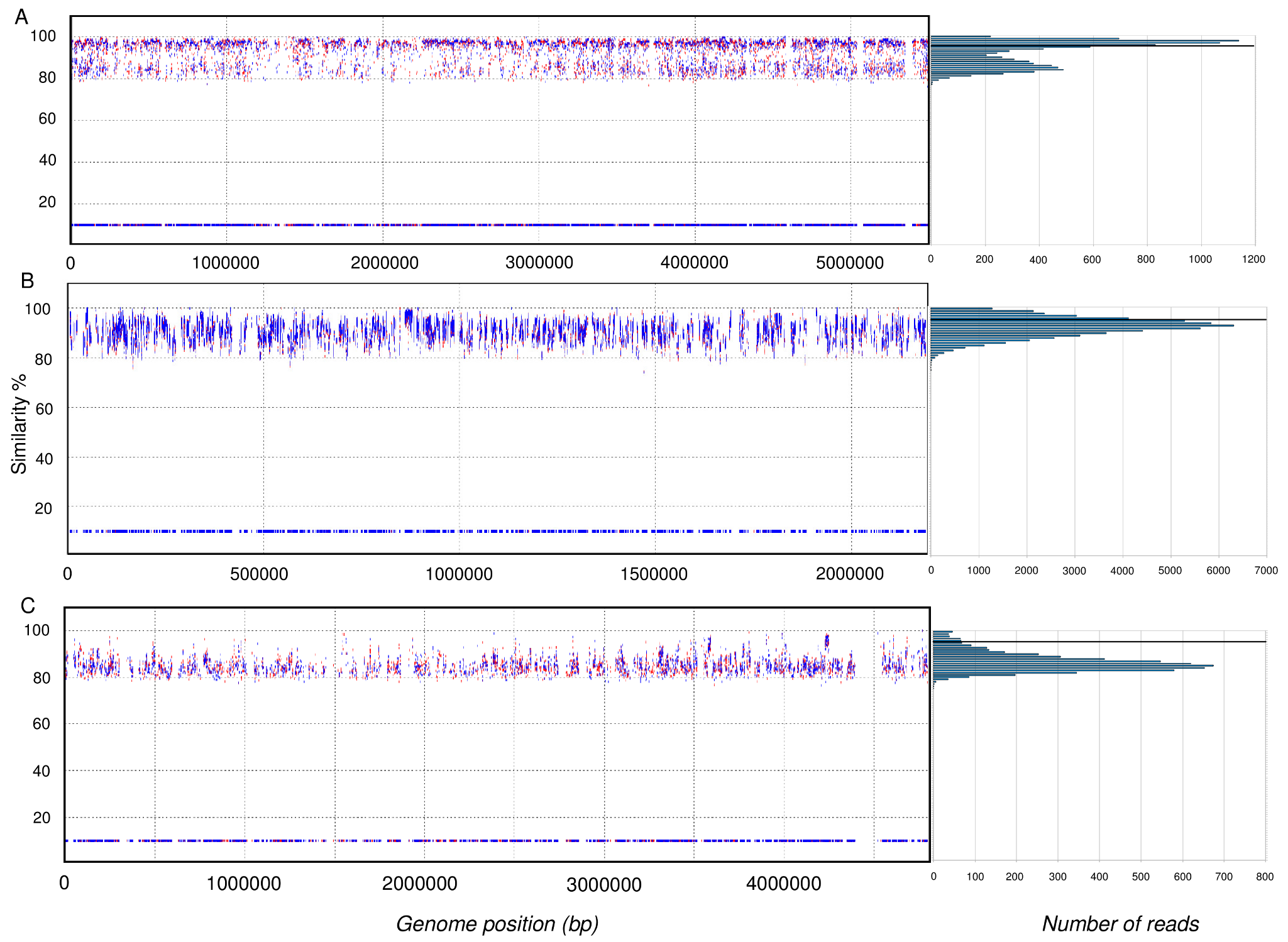

Supplement: Figure S1 — Nucleotide recruitment plots obtained from comparing the intestinal metagenome against the genome of Escherichia coli O157:H7 Sakai str. ( A ) and Salmonella enterica subsp. enterica serovar Typhi str. CT18 (C), and from the oral metagenome against Neisseria meningitidis FAM18 (B). Graphs on the right show frequency histograms of the similarity values of the reads mapped to a given genome. The black line marks the 94% standard threshold for mean identity values for strains from the same species. Taking this line as a threshold, the recruitments are performed against bacteria from the same species as the reference genome (A), different species from the same genus (B) or against a different genus (C). (TIF) [file pone.0024975.s001.tif]
