# Supplementary material for: Mining Virulence Genes Using Metagenomics
Source: PLoS One. 2011 Oct 19;6(10):e24975. doi: 10.1371/journal.pone.0024975 (PMC3198465; doi:10.1371/journal.pone.0024975)
Supplement: Table S1 — Full gene content of Metagenomic Islands detected by recruitment of selected pathogenic species of pathogenic bacteria against the gut and oral metagenomes. (PDF) [file pone.0024975.s002.pdf]

**Supplementary Table 1.**
**Full gene content of Metagenomic Islands detected by recruitment of selected pathogenic species of pathogenic bacteria against the gut and oral metagenomes.**

## *Escherichia coli* E24377A ETEC vs gut metagenome

| Start-End (bp) | Length (kbp) | Number of ORFs | Main features (in brackets number of genes) |
|---|---|---|---|
| 19539-22862 | 3.3 | 2 | hypothetical protein(2) |
| 240344-244042 | 3.7 | 3 | hypothetical protein (2), ImpA domain-containing protein (1) |
| 295666- 306687 | 11.02 | 7 | hypothetical protein (5), prophage CP4-57 regulatory protein (1), SNF2 family helicase (1) |
| 315072- 319208 | 4.13 | 2 | hypothetical protein(1), UvrD family helicase (1) |
| 608338- 614564 | 6.2 | 3 | hypothetical protein (2), YD repeat-containing protein (1); * |
| 698289- 702722 | 4.4 | 6 | hypothetical protein (5), DnaJ domain-containing protein (1) |
| 836672- 843690 | 7.01 | 6 | hypothetical protein (2), multidrug efflux protein, IS66 family transposase, IS66 family orf2, IS66 family orf1 (*) |
| 1000659-1013491 | 12.8 | 13 | phage integrase family site specific recombinase, phage regulatory protein, putative replication gene B protein, C4-type zinc finger DksA/TraR family protein, replication gene A protein, hypothetical protein (5), PBSX family phage portal protein, hypothetical protein, IS21 family transposase, IS21 family transposition helper protein |
| 1063848- 1083251 | 19.4 | 20 | tail fiber assembly protein, putative phage tail fiber protein, hypothetical protein (2), baseplate assembly protein J, phage protein, phage baseplate assembly protein V, bacteriophage Mu P protein, bacteriophage Mu transposase MuA, putative repressor protein, aliphatic sulfonate ABC transporter periplasmic substrate-binding protein, NAD(P)H-dependent FMN reductase, fimbrial protein (3), periplasmic pilus chaperone family protein, outer membrane usher protein fimD, putative fimbrial protein, putativi pili assembly chaperone |
| 1124464- 1128783 | 4.3 | 4 | putative lipoprotein, hypothetical protein, group 4 capsule (G4C) polysaccharide; lipoprotein YmcC, putative inner membrane protein |
| 1399972-1448135 | 48.7 | 50 | phage N-6-adenine-methyltransferase, hypothetical protein (32), IS66 family orfs (3), putative recombinase, transport protein TonB, acyl-CoA thioester hydrolase, intracellular septation protein A, outer membrane protein W, phage integrase recombinase, exonuclease family protein, Rha family phage regulatory protein, DNA-binding transcriptional regulator DicC, crossover junction endodeoxyribonuclease RusA-like protein, phage antitermination protein Q, protein kinase domain-containing protein, DNA methylase, lambda phage portal protein |

| | | | |
|---|---|---|---|
| 1626864- 1635094 | 8.2 | 4 | hypothetical protein (2), type VI secretion system Vgr family protein, protein rhsD, truncation. |
| 1755818- 1761438 | 5.6 | 7 | hypothetical protein, IS66 family transposase (2), IS66 family orf2 (2), IS66 family orf1 (2) |
| 2172994-2227046 | 54.05 | 91 | hypothetical protein (38), tail fiber family protein, major tail sheath protein, phage tail protein I, baseplate assembly protein J, baseplate assembly protein W, phage baseplate assembly protein V, phage major capsid protein E, bacteriophage lambda head decoration protein D, family peptidase, IS21 (2), phage terminase large subunit (GpA), phage antitermination protein Q, lipoprotein (2), DnaB family helicase, transcriptional repressor DicA, phage integrase, invasion, shikimate transporter, AMP nucleosidase, transcriptional regulator Cbl, nitrogen assimilation transcriptional regulator, nicotinate dimethylbenzimidazole phosphoribosyltransferase, cobalamin synthase, adenosylcobinamide kinase/adenosylcobinamide-phosphate guanylyltransferase, cobalamin biosynthesis, regulatory protein PocR, propanediol diffusion facilitator, propanediol utilization protein PduA and B, propanediol dehydratase (18), gyrase inhibitor, D-alanyl-D-alanine carboxypeptidase, exonuclease I, YeeE/YedE, amino acid permease |
| 2283962- 2292498 | 8.5 | 7 | hypothetical protein (4), VI polysaccharide biosynthesis protein vipB/tviC, VI polysaccharide biosynthesis protein VipA/tviB, glycosyl transferase group 2 family protein (2) |
| 2907653- 2949345 | 41.69 | 28 | resolvase family site-specific recombinase, parB family protein, repB plasmid partitioning protein, N4/N6-methyltransferase family protein, putative type I restriction-modification system, S subunit, HsdR family type I site-specific deoxyribonuclease, hypothetical protein (14), relaxase/mobilization nuclease domain-containing protein, putative lipoprotein (2), IS21 family transposase (2), IS66 family (2) |
| 3020396- 3032923 | 12.5 | 7 | hypothetical protein (4), IS3; transposase orfA, IS3; transposase orfB, putative phage integrase family protein |
| 3184041-3202475 | 18.4 | 18 | hypothetical protein (6), transcriptional regulatory protein (C terminal), TPR repeat-containing protein, transcriptional regulator, LuxR family transcriptional regulator, type III secretion apparatus lipoprotein EprK, type III secretion apparatus protein EprH, FlhB/HrpN/YscU/SpaS family protein, type III secretion apparatus proteins EpaR/Q/O2, surface presentation of antigens protein SpaP, type III secretion apparatus protein (truncation). |
| 3328251-3341175 | 12.9 | 8 | hypothetical protein (3), SNF2 family helicase, AAA family ATPase, S8A family peptidase, DNA methylase, type III restriction enzyme, res subunit |
| 3373202-3397803 | 24.6 | 21 | phage integrase family protein, helicase/Zfx/Zfy transcription activation region domain-containing protein, hypothetical protein (8), Ig family protein, UvrD family helicase, IS66 family orf1, IS66 family orf2, IS66 family transposase, IS21 family (2), DnaB family helicase, chromosome partitioning protein, site-specific recombinase, phage integrase family protein. |

| | | | |
|---|---|---|---|
| 3406613-3414091 | 7.4 | 7 | IS66 transposase, IS66 family orf2, IS66 family orf1, DNA-binding protein H-NS-like protein, hypothetical protein, prophage CP4-57 regulatory protein, hypothetical protein |
| 3519564-3521299 | 1.7 | 2 | periplasmic pilus chaperone family protein, hypothetical protein |
| 3575976- 3581426 | 5.4 | 4 | putative pilus biogenesis initiator protein, hypothetical protein (2), putative CS1 type fimbrial major subunit. |
| 3624219- 3629648 | 5.4 | 4 | fimbrial protein, pili assembly chaperone protein, fimbrial usher family protein, putative fimbrial protein. |
| 3942979- 3946457 | 3.5 | 2 | hypothetical protein (2). |
| 4078565-4083334 | 4.8 | 2 | hypothetical protein, RHS domain-containing protein |
| 4263383-4268559 | 5.2 | | Not annotated |
| 4360765-4365797 | 5.03 | | Not annotated |
| 4854251- 4858358 | 4.1 | 8 | iron-dicitrate transporter ATP-binding subunit, iron-dicitrate transporter subunit FecD, iron-dicitrate transporter permease subunit, iron-dicitrate transporter substrate-binding subunit, fec operon regulator FecR, FecI. |
| 4887773- 4898030 | 10.25 | 7 | type III restriction enzyme, res subunit, N4/N6-methyltransferase family protein, hypothetical protein (2) |

## *Escherichia coli* CFT073 vs gut metagenome

| **Start-End (bp)** | **Length (kbp)** | **Number of ORFs** | **Main features (in brackets number of genes)** |
|---|---|---|---|
| 274627-279569 | 4.9 | 3 | hypothetical protein (3) |
| 292801- 298303 | 5.5 | 5 | putative oligogalacturonide transporter, putative exopolygalacturonate lyase, hypothetical protein (3) |
| 315930-320291 | 4.3 | 1 | ShlA/HecA/FhaA exofamily protein |
| 331863-347819 | 15.6 | 13 | hypothetical protein (10), putative cytoplasmic membrane export protein, putative membrane spanning export protein, RTX family exoprotein A gene. |
| 908887-942273 | 33.4 | 48 | integrase, hypothetical protein (22), putative regulator for prophage, DNA adenine methylase, prophage terminase, ATPase subunit, putative capsid scaffolding protein, major capsid protein terminase, endonuclease subunit, putative capsid completion protein, phage tail protein secretory protein, Fels-2 prophage lysozyme, putative regulatory protein, phage tail protein (4), Phage baseplate assembly protein (3), variable tail fibre protein, major tail sheath protein, putative tail fiber protein of prophage (2), putative regulator of late gene expression, prophage P2 Ogr protein. |
| 1127548-1135398 | 7.8 | 6 | P4 family integrase, hypothetical protein (4), prophage CP4-57 regulatory protein alpA |
| 1142842-1163920 | 21.07 | 24 | hypothetical protein (16), 3-oxoacyl-(acyl carrier protein) synthase II, 3-ketoacyl-(acyl-carrier-protein) reductase, hypothetical protein, 3-oxoacyl-(acyl carrier protein) synthase I, acyl carrier protein, putative acyl carrier protein, putative phospholipid biosynthesis acyltransferase, putative O-methyltransferase |

| | | | |
|---|---|---|---|
| 1169911- 1173103 | 3.2 | 4 | hypothetical protein (2), putative transposase, phospho-2-dehydro-3-deoxyheptonate aldolase |
| 1175693-1182738 | 7.04 | 5 | MchB protein, MchC protein, MchD protein, microcin H47 secretion protein (2) |
| 1186790- 1194173 | 7.4 | 10 | putative F1C and S fimbrial switch regulatory protein (2), F1C fimbrial subunit precursor (5), F1C periplasmic chaperone, F1C fimbrial usher |
| 1202703- 1207331 | 4.6 | 1 | ABC transporter ATP-binding protein |
| 1218885- 1224353 | 5.5 | 6 | hypothetical protein (5), antigen 43 precursor |
| 1350647-1353648 | 3 | 4 | putative DNA packaging protein of prophage (terminase large subunit), putative DNA packaging protein of prophage, putative capsid protein of prophage (2, one truncated) |
| 1759666-1763220 | 3.5 | 1-2 | hypothetical protein, (zinc protease pqqL, truncated) |
| 2748603- 2758995 | 10.2 | 4 | yapH-like protein, hypothetical protein, Type 1 fimbriae regulatory protein fimB (2) |
| 2988732- 2992446 | 3.7 | | Not annotated |
| 3234417- 3239912 | 5.5 | 4 | hypothetical protein (4) |
| 3416671-3420519 | 3.8 | 3 | hypothetical protein, hemolysin C, hemolysin A (truncated) *Region 3406225-3450866 corresponds to well characterized Pathogenicity Island I (Alpha-hemolysin, P-fimbriae, aerobactin)* |
| 3432331-3436862 | 4.5 | 4 | PapJ protein, PapD protein, PapC protein, PapH protein. |
| 3452943-3456117 | 3.2 | 4 | hypothetical protein (4) |
| 3464575-3472925 | 8.3 | 9 | IucC protein, IucB protein, IucA protein, shiF protein, hypothetical protein (5) |
| 3520088-3527942 | 7.8 | 5 | hypothetical protein (4), putative glycerol-3-phosphate cytidyltransferase. |

***Salmonella enterica subsp. enterica serovar Typhi str. CT18 vs gut metagenome***

| **Start-End (bp)** | **Length (kbp)** | **Number of ORFs** | **Main features (in brackets number of genes)** |
|---|---|---|---|
| 14807-37104 | 22.3 | 20 | Fimbrial proteins (6), hypothetical protein (9), transcriptional regulator (3), sulfatase (1), chitinase (1) |
| 302188-360345 | 58.1 | 51 | hypothetical protein (35), fimbrial proteins (7), outer membrane adhesin (1), lipoprotein (2) Rhs-family protein (3), transcriptional regulator (2), ClpB-like protein (1) *Region 302092-360757 established as Pathogenicity Island 6. Function : safA-D and tcsA-R chaperone-usher fimbrial operons* |
| 378000-393000 | 15 | 16 | Fimbrial proteins (5), hypothetical protein (5), transmembrane regulator (2), transcriptional regulator (1), lipoprotein (1), outer membrane protein (1). |
| 1008756 -1053000 | 43 | 65 | Putative bacteriophage proteins (33), hypothetical protein (10), putative secreted protein (2),putative DNA-binding protein (3), putative replication protein (1), putative prophage terminase small (1) and large (1) subunit, putative prophage membrane protein (2), putative prophage antitermination protein (1), putative |

| | | | |
|---|---|---|---|
| | | | methyltransferase (1), putative lipoprotein (1), bacteriophage recombination protein (1), excisionase (1), exonuclease (1), FtsZ inhibitor protein (1), host-nuclease inhibitor protein (1), putative damage-inducible protein (1), DNA invertase (1), DNA methylase (1), integrase (1). |
| 1085173-1094111 | 8.9 | 9 | Transposase for insertion sequence element is200, hypothetical protein (4), cell invasion protein (2), putative secreted peptidase, histidine kinase. *Region 1085068-1092563 established as PAI5. Function : Effector proteins for SPI-1 and SPI-2 (SopB, SigD, PipB)* |
| 1465324-1486831 | 21.5 | 18 | hydrogenase 1 maturation protease (1), hydrogenase isoenzyme formation protein (1), hydrogenase-1 operon protein HyaE2 (1), hydrogenase-1 operon protein HyaF2 (1), hypothetical protein (2), membrane transport protein (1), Ni/Fe-hydrogenase 1 b-type cytochrome subunit HyaC2 (1), putative alcohol dehydrogenase (1), putative aminotransferase (1), putative ATP/GTP-binding protein (1), putative isomerase (1), putative multidrug efflux protein (1), putative regulatory protein (2), putative secreted hydrolase (1), putative transport protein (1), uptake hydrogenase small subunit (1). |
| 1625104-1650621 | 25.5 | 32 | putative outer membrane secretory protein (1), putative pathogenicity island 2 secreted effector protein (7), putative pathogenicity island lipoprotein (1), putative pathogenicity island protein (9), putative secretion system protein (2), two-component response regulator (1), two-component sensor kinase (1), putative type III secretion protein (4), Type III secretion system chaperone protein (1), secretion system apparatus protein SsaU (1), secretion system apparatus protein SsaV (1), type III secretion system ATPase (1), type III secretion system protein (2). *Region 1624920-1666524 established as PAI2. Function : Type III secretion system, required for systemic infection and intracellular pathogenesis. Insertion site : tRNA-val* |
| 1768224-1791992 | 23.8 | 33 | Hypothetical protein (13), lysozyme inhibitor, outer membrane invasion protein, putative ABC transport ATP-binding (2), putative bacteriophage protein, putative cold shock protein, putative cytochrome, putative heat shock protein, putative inner membrane transport protein (2), putative lipoprotein (3), putative outer membrane virulence protein, substrate-binding transport protein, putative toxin-like protein, putative virulence protein, toxin subunit (2), transposase for IS200. |
| 1818448- 1930625 | 112.2 | 131 | 23S rRNA methyltransferase A, alanine racemase, carboxy-terminal protease, cell division inhibitor MinD, cell division topological specificity factor MinE, cold shock-like protein CspC, D-amino acid dehydrogenase small subunit, exonuclease VIII, fatty acid metabolism regulator, FtsZ inhibitor protein, heat shock protein HtpX, host cell-killing modulation protein, hypothetical protein (58), L-serine deaminase 1, L,D-carboxypeptidase A, long-chain-fatty-acid--CoA ligase, mannose-specific PTS system protein IID, membrane-bound lytic murein transglycosylase E, para-aminobenzoate synthase component I, penicillin-binding protein, phosphotransferase enzyme II C component, |

| | | | |
|---|---|---|---|
| | | | potassium/proton antiporter, PTS system mannose-specific IIAB component, putative acetyltransferase, putative bacteriophage protein (31), bacteriophage tail protein (2), putative Cro repressor, DNA-binding protein, putative endolysin, putative hydrolase, lipoprotein, putative regulator, replication protein, solute/DNA competence effector, transposase, ribonuclease D, RsmF, septum formation inhibitor, serine/threonine protein phosphatase 1, sodium/proton antiporter, SpoVR family protein, transcriptional regulator KdgR. |
| 2742887-2800000 | 57.2 | 42 | Large repetitive protein, putative type I secretion protein (2), putative secretion protein ATP-binding protein, 4-aminobutyrate aminotransferase, DNA binding protein nucleoid-associated, DNA-binding transcriptional regulator CsiR, gamma-aminobutyrate transporter, hydroxyglutarate oxidase, hypothetical protein (12), major tail tube protein, outer membrane receptor FepA, putative ABC transporter protein, putative bacteriophage late gene regulator (2), putative bacteriophage major tail sheath protein, putative bacteriophage (2), putative ferric enterochelin esterase, putative transcriptional regulator (2), two-component system sensor kinase, putative type I secretion protein, succinate-semialdehyde dehydrogenase I, transcriptional regulator, virulence protein.<br>*Region 2743495-2759190 established as PAI9. Function : Type I secretory apparatus, including large RTX-like protein* |
| 2862867-2900000 | 37.1 | 38 | acyl carrier protein, AraC family transcription regulator (4), ATP synthase SpaL, cell adherance/invasion protein (5), chaperone (associated with virulence), hypothetical protein (5), invasion protein regulator, pathogenicity 1 island effector protein (8), secretory protein (associated with virulence) (6), serine/threonine-specific protein phosphatase 2, surface presentation of antigens protein SpaO/SpaP/SpaS(associated with type III secretion and virulence), tyrosine phosphatase (associated with virulence) (4).<br>*Region 2858736-2900586 established as PAI-1. Function : Type III secretion system, invasion into epithelial cells, apoptosis (InvA, OrgA, SptP, SipA, SipB, SipC, SipD, SopE, prgH). Insertion site : fhlA/mutS* |
| 3042054-3059937 | 17.9 | 17 | endonuclease fragment, fimbrial chaperone protein, fimbrial protein, hypothetical protein (10), integrase, outer membrane fimbrial usher protein, outer membrane protein (associated with virulence), plasmid maintenance. |
| 3133624-3139922 | 6.3 | 12 | Hypothetical protein (10), bacteriocin immunity protein.<br>*Region 3132530-3139414 established as PAI8. Function : Two bacteriocin pseudogenes, genes conferring immunity to the bacteriocins. Insertion site : tRNA-phe* |
| 3515395-3549044 | 33.6 | 47 | bacteriophage integrase, capsid portal protein, DNA adenine methylase, DNA-invertase, endonuclease, hypothetical protein (15), lipoprotein (2), major capsid protein, major tail sheath protein, major tail tube protein, phage baseplate assembly protein (2), phage tail protein, putative capsid completion protein, putative capsid |

| | | | |
|---|---|---|---|
| | | | scaffolding protein, putative lysozyme, phage tail protein (5), putative positive regulator of late gene transcription (2), putative regulatory protein, regulatory protein cII, repressor protein, secretory protein, terminase ATPase subunit, terminase endonuclease subunit, transposase, variable tail fibre protein. |
| 3889742-3897176 | 7.4 | 3 | Hypothetical protein (2), putative DNA binding protein. Region 3883613-3900553 established as PAI3. Function: Invasion, survival in monocytes, Mg2+ uptake. Insertion site : tRNA-pro. |
| 4321946-4346916 | 25 | 9 | hypothetical protein (2), large repetitive protein (2), putative integral membrane protein, putative type-1 secretion protein (3), single-stranded DNA-binding protein. *Region 4322993-4346383 established as PAI-4. Function: Type I secretion system, putative toxin secretion, apoptosis, required for intracellular survival in macrophages, genes weakly similar to RTX-like toxins.* |
| 4402991-4543069 | 140 | 153 | AraC family transcription regulator, bacteriophage integrase, capsid portal protein, DNA adenine methylase, DNA helicase, DNA polymerase V subunit UmuC, DNA topoisomerase III,GerE family regulatory protein, hypothetical protein (86), IS1 (2), integrase (fragment), invasion-associated secreted protein, tail sheath protein, major tail tube protein, nonspecific acid phosphatase precursor, nucleotide-binding protein, phage baseplate assembly (2), phage integrase (2), phage regulatory protein (2), phage protein (3), PilN lipoprotein, pilus assembly protein, prepilin (4), putative acetyltransferase, capsid completion protein, putative capsid protein, DNA helicase, putative exonuclease, putative lipoprotein, lysozyme, major capsid protein, methyltransferase, phage baseplate assembly protein, phage tail protein (7), phage terminase, pilus assembly protein, positive regulator of late gene transcription (2), regulatory protein, secretion protein, shufflon-specific DNA recombinase, single-stranded DNA-binding protein, terminase subunit, transcriptional regulatory protein, VI polysaccharide biosynthesis protein (2), VI polysaccharide protein (10). *Region 4409511-4543148 established as PAI7. Function : Vi exopolysaccharide, SopE prophage and a type IVB pilus operon. Insertion site : tRNA-phe* |

## *Escherichia coli* O157:H7 str Sakai vs gut metagenome

| **Start-End (bp)** | **Length (kbp)** | **Number of ORFs** | **Main features (in brackets number of genes)** |
|---|---|---|---|
| 18284-24143 | 5.9 | 7 | hypothetical protein (4), putative outer membrane usher protein precursor, putative fimbrial protein (2) |
| 154305-161555 | 7.3 | 7 | putative fimbrial related protein (7) |
| 226821-231340 | 4.5 | 1 | putative phosphatase |
| 311962-315433 | 3.5 | 6 | hypothetical protein (6) |
| 579634-605089 | 25.5 | 5 | hypothetical protein (2), putative outer membrane transport protein, putative ATP-binding component of a transport system, putative membrane fusion protein of a |

| | | | |
|---|---|---|---|
| | | | transport system |
| 616665-620312 | 3.6 | 1 | RhsD core protein with extension |
| 659088-663506 | 4.4 | 2 | bacteriophage N4 adsorption protein Nfr (2) |
| 665897-673709 | 7.8 | 4 | Rhs core protein (2), hypothetical protein (2) |
| 756972-761392 | 4.4 | 5 | hypothetical protein (3), putative enzyme of polynucleotide modification, putative tRNA ligase |
| 808998-813939 | 4.9 | 2 | RhsC core protein with extension, hypothetical protein |
| 824993-827911 | 2.9 | 3 | hypothetical protein, putative chaperone, putative outer membrane protein |
| 896902-901800 | 4.9 | 6 | NinG protein, serine/threonin protein phosphatase, putative outer membrane protein, antitermination protein, hypothetical membrane protein, hypothetical protein |
| 925931-929688 | 3.8 | 4 | Hypothetical protein (4) |
| 1130621-1134072 | 3.5 | 5 | hypothetical protein (3), homolog of Salmonella FimH protein, putative fimbrial-like protein. |
| 1161155-1165787 | 4.6 | 5 | putative integrase, hypothetical protein (3), putative division inhibition protein. |
| 1180375-1182095 | 1.7 | 1 | hypothetical protein |
| 1185506-1189603 | 4.1 | 8 | hypothetical protein (7), putative holin protein. |
| 1189827-1196688 | 6.9 | 7 | Phage related protein (6), hypothetical protein. |
| 1266910-1268457 | 1.5 | 3 | Hypothetical protein, Shiga toxin 2 subunit A and B |
| 1275281-1285769 | 10.5 | 11 | hypothetical protein (7), phage related protein (4) |
| 1287067-1306278 | 19.2 | 17 | hypothetical protein (12), putative outer membrane protein (3), putative tail tip fiber protein, MokW protein. |
| 1321735-1324060 | 2.3 | 1 | hypothetical protein |
| 1337169-1361748 | 24.6 | 18 | FidL-like protein, hemagglutinin related protein (2), |
| 1365418-1378122 | 12.7 | 8 | hypothetical protein (4), putative integrase, putative membrane protein, transposase, putative regulatory protein |
| 1403704-1409766 | 6.1 | 9 | Hypothetical protein (8), TerW protein |
| 1412093-1415215 | 3.1 | 4 | putative tellurium resistance protein (4) |
| 1420952-1424909 | 4 | 6 | hypothetical protein (6) |
| 1544512-1549614 | 5.1 | 11 | hypothetical protein (9), putative phage related protein (2) |
| 1561435-1575771 | 14.3 | 15 | Putative phage related protein (12), hypothetical prots. (3) |
| 1579575-1585557 | 6 | 3 | putative secreted effector protein, hypothetical protein (2) |
| 1601708-1608895 | 7.2 | 11 | Putative phage related protein (7), hypothetical protein (4) |
| 1766113-1769281 | 3.2 | 3 | putative fimbrial minor pilin protein precursor (2), putative colonization factor. |
| 1774074-1789217 | 15.1 | 19 | hypothetical protein (8), putative holin protein, putative endolysin, antirepressor protein, endopeptidase, lipoprotein Rz1 precursor, putative Dnase, putative phage related protein (5) |

| | | | |
|---|---|---|---|
| 1790629-1794373 | 3.7 | 3 | Phage related protein (3) |
| 1800640-1803498 | 2.9 | 3 | hypothetical protein (3) |
| 1803739-1811589 | 7.9 | 12 | Integrase, hypothetical protein (11) |
| 1922707-1925718 | 3 | 5 | hypothetical protein (2), restriction alleviation and modification enhancement protein, recombinase recT, exonuclease VIII RecE |
| 1934311-1938964 | 4.7 | 4 | hypothetical protein (3), putative methyltransferase. |
| 1940951-1943871 | 2.9 | 3 | Hypothetical protein. |
| 1944553-1956524 | 12 | 17 | putative endolysin, putative antirepressor protein, putative endopeptidase, hypothetical protein (7), putative Dnase, putative phage related protein (6), |
| 1957922-1962025 | 4.1 | 3 | Phage related protein (3) |
| 2042654-2050534 | 7.9 | 3 | hypothetical protein, VgrE protein, RhsE core protein |
| 2095238-2098792 | 3.6 | 1 | hypothetical protein |
| 2116542-2122543 | 6 | 2 | putative ATP-binding component of a transport system and adhesin protein, hypothetical protein |
| 2158654-2165353 | 6.7 | 8 | hypothetical protein (4), phage related protein (4) |
| 2176148-2179103 | 3 | 3 | Phage related protein (3) |
| 2184405-2190561 | 6.2 | 6 | putative endolysin, putative holin protein, putative transcriptional regulator, hypothetical protein (3) |
| 2218918-2222988 | 4.1 | 1 | putative tail length tape measure protein |
| 2224586-2241664 | 17.1 | 24 | Hypothetical protein (12), endopeptidase, lipoprotein Rz1 precursor, putative antirepressor protein, putative antitermination protein, putative Dnase, putative endolysin, putative head-tail adaptor, putative holin protein, putative major head protein/prohead protease, putative portal protein, putative terminase large subunit, putative terminase small subunit. |
| 2484600-2487761 | 3.2 | 2 | putative transport protein, hypothetical protein. |
| 2668068-2670857 | 2.8 | 3 | Hypothetical protein (2), EspF-like protein |
| 2673153-2688564 | 15.4 | 17 | Host specificity protein, hypothetical protein, phage related protein (15) |
| 2694791-2698923 | 4.1 | 5 | Hypothetical protein (4), antiterminator. |
| 2714118-2718695 | 4.6 | 1 | Putative factor |
| 2780835-2787527 | 6.7 | 6 | putative glycosyl transferase (3), perosamine synthetase, O antigen flippase, O antigen polymerase |
| 2901449-2905616 | 4.2 | 5 | Putative phage related protein (5) |
| 2924228-2926965 | 2.7 | 4 | Shiga toxin I precursor (2), antitermination protein, hypothetical protein. |
| 3075378-3079251 | 3.9 | 4 | putative antibiotic resistance protein, putative transcriptional regulator, hypothetical protein, glycerophosphodiester phosphodiesterase |
| 3478307-3480077 | 1.8 | 2 | hypothetical protein (2) |
| 3492963-3498985 | 6 | 6 | hypothetical protein (4), putative site specific recombinase, |

| | | | |
|---|---|---|---|
| | | | putative DNA binding protein |
| 3505135-3511008 | 5.9 | 4 | Hypothetical protein (4) |
| 3709749-3737163 | 27.4 | 35 | Hypothetical protein (15), putative invasion protein, putative sensory transducer, putative transcriptional regulator, tyoe III secretion system protein (17) |
| 3866707-3872713 | 6 | 7 | putative adherence factor (2), transposase (2), hypothetical protein (3) |
| 4159436-4164182 | 4.7 | 0 | Not annotated |
| 4365737-4369236 | 3.5 | 3 | Hypothetical protein (3) |
| 4585467-4605785 | 20.3 | 25 | CesT protein, Esc protein, Esp protein (4), gamma intimin, hypothetical protein (14), translocated intimin receptor, type III secretion system protein(3) |
| 4606989-4609848 | 2.9 | 4 | Type III secretion system protein(3), hypothetical protein |
| 4610632-4614888 | 4.3 | 4 | type III secretion system protein (4), hypothetical prot. (2) |
| 4615973-4624447 | 8.5 | 14 | Type III secretion system protein(4), hypothetical protein (8), Ler protein, EspG protein |
| 4686222-4693486 | 7.3 | 7 | Hypothetical protein (7) |
| 4928827-4931803 | 3 | 1 | RhsH core protein with extension |
| 4975790-4979751 | 4 | 0 | Not annotated |
| 5017602-5022018 | 4.4 | 0 | Not annotated |
| 5028745-5031187 | 2.4 | 1 | regulator of acetyl CoA synthetase |
| 5057479-5075494 | 18 | 22 | Hypothetical protein (11), phage related protein (11). |
| 5359996-5367261 | 7.3 | 5 | hypothetical protein (4), putative membrane protein. |
| 5382504-5391510 | 9 | 4 | putative RNA helicase, putative DNA helicase, hypothetical protein (2) |
| 5416303-5421927 | 5.6 | 3 | putative invasin, hypothetical protein (2) |

## *Shigella flexneri* 2a str. 301 vs gut metagenome

| **Start-End (bp)** | **Length (kbp)** | **Number of ORFs** | **Main features (in brackets number of genes)** |
|---|---|---|---|
| 214885-219996 | 5.1 | 0 | Not annotated |
| 229909-244882 | 15 | 19 | Hypothetical protein (2), IS2 transposase InsD, insertion sequence 2 OrfA protein, IS911 (3), outer membrane usher protein, periplasmic chaperone of fimbral assembly machinery, coat protein, cytoplasmic protein (5), putative DNA stabilization protein, packaging glycoprotein, putative scaffolding protein, putative terminase large subunit. |
| 262348-267750 | 5.4 | 9 | Hypothetical protein (3), insertion element IS2 transposase InsD, insertion sequence 2 OrfA protein, IS1 (2), IS600 (2). |
| 278203-287655 | 9.5 | 8 | Hypothetical prots. (2), IS1 ORF (2), Rhs-family protein (4). |
| 311516-332269 | 20.8 | 26 | Hypothetical protein (7), integrase, IS600 ORF (8), IS629 ORF (3), putative bactoprenol glucosyl transferase, putative flippase, putative glucosyl tranferase II, putative phage integrase (3), putative phage tail fibre protein. |

| | | | |
|---|---|---|---|
| 374550-380512 | 6 | 5 | Hypothetical protein (4), IS1 ORF. |
| 511825-512589 | 0.8 | 1 | phosphopantetheinyltransferase component of enterobactin synthase multienzyme complex |
| 618107-624102 | 6 | 6 | Hypothetical protein (2), Rhs-family protein (2), IS1 (2). |
| 699193-750156 (low coverage region) | 51 | 60 | Capsid protein small subunit, endopeptidase, head-tail preconnector gp5 (3), host specificity protein, hypothetical protein (3), insertion element IS2, insertion sequence 2 OrfA protein, invasion plasmid antigen, IS600 ORF (7), IS629 ORF (3), IS911 ORF (4), major capsid protein, minor tail protein, putative bacteriophage protein (19), putative Q protein, putative replication protein DnaC, putative S protein, putative tail assembly protein, putative tail attachment protein, putative tail component, putative tail component of prophage CP-933K (7), putative tail length tape measure protein precursor. |
| 898345-921546 | 23.2 | 28 | DNA-binding transcriptional regulator DicC, hypothetical protein (6), insertion element IS2 transposase InsD, insertion sequence 2 OrfA protein, IS2 ORF2, IS600 ORF (6), IS911 ORF (2), ISSfl4 ORF (3), putative bacteriophage protein (5), putative exodeoxyribonuclease VIII of prophage CP-933R, transcriptional repressor DicA. |
| 984277-991700 | 7.4 | 9 | Fimbrial protein, hypothetical protein, IS1 ORF (3), putative fimbrial-like protein (2), putative outer membrane protein, putativi pili assembly chaperone. |
| 1034215-1038587 | 4.4 | 4 | Hypothetical protein (3), putative regulator. |
| 1090857-1103061 | 12.2 | 15 | Hypothetical protein (3), insertion element IS2 transposase InsD, insertion sequence 2 OrfA protein, IS600 ORF (2), IS629 ORF (4), IS911 ORF (4). |
| 1181034-1188414 | 7.4 | 9 | Hypothetical protein (4), putative head maturation protease of prophage CP-933C, putative head portal protein, putative head-tail adaptor, putative holin protein of prophage CP-933C, putative terminase of prophage CP-933C. |
| 1390300-1395946 | 5.6 | 8 | Hypothetical protein, insertion element IS2 transposase InsD, insertion sequence 2 OrfA protein, IS600 ORF (2), putative bacteriophage protein (2), putative replication protein. |
| 1399435-1405421 | 6 | 8 | putative bacteriophage protein (4), IS600 ORF (2), hypothetical protein (2) |
| 1420617-1425133 | 4.5 | 5 | IS ORF (3), hypothetical protein, invasion plasmid antigen. |
| 1626770-1634439 | 7.7 | 9 | Hypothetical prot. (5), IS911 ORF (2), putative integrase (2) |
| 1917457-1941923 | 24.4 | 25 | Phage /mobile elements island<br>Host specificity protein, invasion plasmid antigen, IS1 ORF (2), IS600 ORF (2), ISSfl2 ORF, minor tail protein (8), putative crossover junction endodeoxyribonuclease, putative DNA-packaging protein, putative membrane protein precursor, putative Q antiterminator encoded by prophage CP-933P, putative serine protease, putative tail component of prophage CP-933K (6), putative tail length tape measure protein precursor. |
| 2044876-2053192 | 8.3 | 7 | Hypothetical protein (3), IS1 ORF2, IS600 ORF2, iso-IS10R ORF, putative tail protein. |

| | | | |
|---|---|---|---|
| 2107750-2115293 | 7.5 | 9 | LPS SYNTHESIS ISLAND<br>dTDP-rhamnosyl transferase (2), glycosyl transferase, glycosyl translocase, hypothetical protein (3), O-antigen polymerase, polysaccharide biosynthesis protein. |
| 2227913-2236010 | 8.1 | 9 | DNA-damage-inducible protein, hypothetical protein (3), IS1 ORF (2), iso-IS10R ORF, putative tail fiber assembly protein, putative tail fiber protein. |
| 2552420-2557999 | 5.6 | 6 | Hypothetical protein (6). Between genes of peptydoglycan synthesis. |
| 2589066-2598121 | 9.1 | 7 | Hydrogenase 4 Fe-S subunit, hydrogenase 4 membrane subunit, hydrogenase 4 subunit D, hydrogenase 4 membrane subunit, hydrogenase 4 subunit F, large subunit of hydrogenase 3 (2). |
| 2684580-2696870 | 12.3 | 15 | DNA-invertase, hypothetical protein (4), insertion element IS2 transposase InsD, insertion sequence 2 OrfA protein, invasion plasmid antigen, IS600 ORF (2), putative bacteriophage protein, putative tail component of prophage CP-933K, putative tail fiber assembly protein, putative tail fiber protein (2). |
| 2754519-2761442 | 6.9 | 5 | Integrase, IS1 ORF (2), IS3 ORF (2). |
| 2831678-2836482 | 4.8 | 5 | IS600 ORF (2), putative DNA-binding protein, putative phage transposase, putative regulatory protein. |
| 2947609-2957653 | 10 | 11 | Hypothetical protein (5), IS3 ORF (2), IS911 ORF (3), integrase. |
| 3069582-3070249 | 0.7 | 2 | Shet1A, Shet1B<br>*Region 3042550-3097662 corresponds to well established PAI1. It shows good recruitment with small islands, which correspond exactly to regions where pathogenic genes are annotated* |
| 3099253-3107662 | 8.4 | 11 | Hypothetical protein (4), IS1 (4), IS3 (2), IS10R ORF. |
| 3277113-3284686 | 7.6 | 7 | Insertion sequence 2 OrfA protein, IS1 ORF (2), IS2 ORF, putative chaperone, putative fimbrial protein, putative IS2 |
| 3464484-3475679 (low recruitment) | 11.2 | 10 | DNA-binding transcriptional regulator FrlR, fructoselysine 3-epimerase, fructoselysine 6-kinase, fructoselysine-6-P-deglycase, hypothetical protein (2), nitrite reductase (NAD(P)H) subunit, nitrite reductase small subunit, nitrite reductase, NirC protein, siroheme synthase. |
| 3592829-3613524 | 20.7 | 20 | Hypothetical protein (11), insertion element IS2 transposase InsD, insertion sequence 2 OrfA protein, IS1 ORF (2), putative ATP-binding component of a transport system, putative IS1 encoded protein (2), putative outer membrane pore protein, putative periplasmic binding transport protein. |
| 3812410-3836346 | 23.9 | 22 | ColV-immunity protein, hypothetical protein (3), insertion element IS2 transposase InsD (2), insertion sequence 2 OrfA protein (2), IS1 ORF (3), IS629 ORF (2), lysine:N6-hydroxylase, putative ferric siderophore receptor, putative fimbrial protein, putative long polar fimbriae, putative membrane transport protein, serine protease, siderophore biosynthesis protein (3).<br>*Region 3806404-3835200 established as PAI2. Function : Iron uptake systems, immunity to colicin V (IucA, IucB, IucC, IucD, IutA, aerobactin). Insertion site : tRNA-selC* |

| | | | |
|---|---|---|---|
| 4047837-4053555 | 5.7 | 1 | Protoporphyrinogen oxidase, 5 kb with nothing anotated. |
| 4185519-4191708 | 6.2 | 1 | Glutamate racemase, 6 kb with nothing anotated. |
| 4226640-4232128 | 5.5 | 0 | Not annotated |
| 4410160-4414498 | 4.3 | 8 | Hypothetical protein (4), IS1 ORF (2), oxidoreductase, pyrBI operon leader peptide. |
| 4530286-4535960 | 5.7 | 9 | 30S ribosomal protein S18, 50S ribosomal protein L9, endoribonuclease SymE, hypothetical protein (3), IS600 ORF (2), ISEhe3 orfB. |

## *Streptococcus sanguinis* SK36 vs oral metagenome

| **Start-End (bp)** | **Length (kbp)** | **Number of ORFs** | **Main features (in brackets number of genes)** |
|---|---|---|---|
| 16589-22064 | 5.5 | 0 | Not annotated |
| 143437-169792 | 26.4 | 23 | ATPase with chaperone activity ATP-binding subunit putative, Conserved hypothetical protein (9), Cro-like transcriptional repressor XRE family putative, Hypothetical protein (11), Uncharacterized protein |
| 392676- 398388 | 5.7 | 4 | Conserved hypothetical protein (4) |
| 708764-716005 | 7.2 | 9 | Putative ABC-type multidrug/protein/lipid transport system (pediocin PA-1 exporter) ATPase and permease components, putative Arsenical resistance operon transcription repressor (ArsR), putative FmtA-like protein, Hypothetical protein (2), putative Integral membrane protein, putative metal-dependent membrane protease, putative protease, putative transposase. |
| 807557-813010 | 5.4 | 2 | Platelet-binding glycoprotein, putative glycosyltransferase. |
| 1114985-1123882 | 8.9 | 3 | putative calcium binding hemolysin-like protein, putative hemolysin exporter ATPase component, multidrug resistance efflux pump/hemolysin secretion transmembrane protein. |
| 1168936-1179877 | 10.9 | 9 | Putative beta-glucosides PTS (2), beta-N-acetylhexosaminidase, conserved hypothetical protein, dihydrolipoamide acetyl transferase E2 component, glycosyl hydrolase family 1, putative Na+-driven multidrug efflux pump, phosphotransferase system, cellobiose-specific component IIA, tautomerase. |
| 1306316-1310922 | 4.6 | 4 | Conserved hypothetical protein (4) |
| 1631616-1639447 | 7.8 | 5 | Putative sortase-like protein, putative Surface protein, putative, imA fimbrial subunit-like protein, Heme utilization/adhesion exoprotein, Hypothetical protein |
| 1742320-1747543 | 5.2 | 8 | Conserved uncharacterized protein, hypothetical protein (6), putative transcriptional regulator GntR family (repressor of trehalose operon). |
| 1796947-1806223 | 9.3 | 7 | Conserved hypothetical protein (4), hypothetical protein, putative modification methylase, putative very short patch repair endonuclease. |
| 1880884-1888335 | 7.5 | 5 | Conserved hypothetical protein (4), putative acetyltransferase. |

| | | | |
|---|---|---|---|
| 1975960-1981997 | 6 | 2 | Putative cell surface SD repeat antigen precursor, conserved hypothetical protein. |
| 2083756-2092917 | 9.1 | 10 | Putative acetyltransferase, putative carbohydrate isomerase AraD/FucA family, putative carbohydrate kinase FGGY family, putative conserved hypothetical protein (5), putative glyoxylate reductase NADH-dependent, putative phosphotransferase system (PTS) galactitol-specific IIC component. |
| 2172522-2178059 | 5.5 | 5 | Hypothetical protein (4), putative 2,3,4,5-tetrahydropyridine-2-carboxylate N-succinyltransferase. |

## *Streptococcus pneumoniae R6 vs oral metagenome*

| **Start-end (bp)** | **Length (kbps)** | **Number of ORFs** | **Main features: genes (in brackets, number of genes)** |
|---|---|---|---|
| 14358-19982 | 5.5 | 0 | Not annotated |
| 29729-30521 | 0.8 | 2 | degenerate transposase (orf1), hypothetical protein |
| 81093-83757 | 3.7 | 1 | cell wall surface anchor family protein |
| 113103-118087 | 5 | 5 | transporter, truncation (2), hypothetical protein (3) |
| 118343-126749 | 8.4 | 6 | Hypothetical proteins |
| 127111-130450 | 3.3 | 10 | hypothetical proteins |
| 140019-149379 | 9.3 | 8 | degenerate transposase (3), glycosyltransferase involved in exopolysaccharide (EPS) synthesis, glycosyl transferase family protein, ABC transporter ATP-binding protein, hypothetical protein<br>UDP-glucose dehydrogenase |
| 218390-221322 | 3 | 4 | DEOR-type transcriptional regulator, hypothetical protein, PTS system, IIA and B components |
| 275026-281685 | 6.6 | 8 | 6-phospho-beta-glucosidase, hypothetical protein, cellobiose phosphotransferase system IIB, C and A components, BigG family transcription antiterminator, hypothetical protein (2) |
| 296522-300559 | 4 | 5 | preprotein translocase, YajC subunit, hypothetical protein, GalR family transcription regulator, hypothetical protein (2) |
| 314511-318935 | 4.4 | 4 | hypothetical proteins |
| 323723-325132 | 1.4 | 3 | hypothetical proteins |
| 349704-351435 | 1.7 | 3 | DNA alkylation repair enzyme, truncated, hypothetical protein, choline binding protein G, truncated, choline binding protein G |
| 354576-360056 | 5.5 | 7 | PTS system, mannitol-specific IIBC components, transcriptional regulator, mannitol-specific enzyme IIA component, mannitol-1-phosphate 5-dehydrogenase, hypothetical protein (2), trigger factor |
| 415208-425429 | 10.2 | 12 | hypothetical protein (3), ROK family protein, PTS system, cellobiose-specific IIC component, hypothetical protein, PTS system, lactose-specific IIA component, 6-P-beta-galactosidase phosphotransferase system sugar-specific EII |

| | | | |
|---|---|---|---|
| | | | component |
| 451863-454743 | 2.9 | 3 | type I restriction-modification system S subunit (2), integrase/recombinase |
| 454834-458104 | 3.3 | 2 | type I restriction-modification system, M and R subunits |
| 473077-474055 | 1 | 2 | hypothetical proteins |
| 474868-476332 | 1.5 | 2 | hypothetical proteins |
| 496875-503646 | 6.8 | 9 | degenerate transposase (2), hypothetical protein (5), BglG family transcriptional antiterminator, PTS system, beta-glucosides-specific IIABC components |
| 539862-542483 | 2.6 | 3 | tributyrin esterase, Serine/alanine adding enzyme, beta-lactam resistance factor |
| 556171-558361 | 2.2 | 0 | Not annotated |
| 591533-593553 | 2 | 2 | Zinc metalloprotease |
| 611637-614204 | 2.6 | 3 | hypothetical proteins |
| 618769-622463 | 3.7 | 4 | hypothetical protein (2), HesA/MoeB/ThiF family protein ABC transporter ATP-binding protein - unknown substrate |
| 622971-624168 | 1.2 | 2 | hypothetical protein, degenerate transposase |
| 625795-629397 | 3.6 | 6 | putative ribonuclease BN, cytochrome c-type biogenesis protein CcdAhypothetical protein, hypothetical protein (3), ABC transporter ATP-binding protein |
| 651680-653048 | 1.4 | 2 | degenerate transposase, hypothetical protein |
| 698647-700072 | 1.4 | 2 | hypothetical proteins |
| 719596-721865 | 2.3 | 3 | transposases |
| 733869-735940 | 2 | 3 | hypothetical protein (2), hemolysin-related protein |
| 756396-757933 | 1.5 | 3 | ABC transporter substrate-binding protein - oligopeptide transport, internal deletion, hypothetical protein (2) |
| 804421-808057 | 3.5 | 6 | hypothetical protein (5), degenerate transposase (orf1) |
| 820142-821585 | 1.4 | 3 | degenerate transposase (2), spermidine synthase |
| 838923-841643 | 2.7 | 3 | hypothetical protein (4), tranposase (orf1 and 2) |
| 852353-854751 | 2.4 | 3 | hypothetical protein (2), ABC transporter ATP-binding protein |
| 886374-888345 | 2 | 5 | degenerative transposase (2), hypothetical proteins |
| 893052-899548 | 6.5 | 4 | pneumococcal histidine triad protein D and E precursor, hypothetical protein, pneumococcal histidine triad protein E precursor, truncation |
| 903628-904825 | 1.2 | 2 | GtrA family protein, hypothetical protein |
| 918685-927808 | 9.1 | 10 | hypothetical protein, iron compound-binding protein, iron-compound ABC transporter, permease protein (2), iron-compound ABC transporter, ATP-binding protein |
| 936137-941289 | 5.1 | 7 | hypothetical protein (2), Tn5252 ORFs (4), putative positive transcriptional regulator MutR |
| 941758-955165 | 13.4 | 9 | UDP-N-acetyl-D-mannosaminuronic acid dehydrogenase, |

| | | | |
|---|---|---|---|
| | | | hypothetical proteins (3), nikkomycin biosynthesis protein, carboxylase, ABC transporter membrane-spanning permease - macrolide efflux |
| 971190-973769 | 2.6 | 4 | degenerate transposase (3) |
| 1052960-1054225 | 1.3 | 2 | hypothetical proteins |
| 1056785-1058377 | 1.6 | 1 | pneumococcal histidine triad protein A precursor |
| 1066637-1068010 | 1.4 | 1 | 6-phospho-beta-galactosidase |
| 1069773-1071118 | 1.3 | 2 | PTS system, lactose-specific IIBC and A component |
| 1087626-1090825 | 3.2 | 2 | hypothetical proteins |
| 1099892-1102232 | 2.3 | 1 | type II restriction endonuclease, putative |
| 1152329-1155285 | 3 | 2 | polysaccharide biosynthesis protein, putative, required for phosphorylcholine incorporation in teichoic and lipoteichoic acids, licD protein, carbamoyl phosphate synthase large subunit |
| 1184169-1187331 | 3.2 | 3 | N-acetylneuraminate lyase subunit, truncation, cytidine deaminase, hypothetical protein (2), ABC transporter ATP-binding protein |
| 1189233-1195071 | 5.8 | 6 | ABC transporter membrane-spanning permease - oligopeptide transport (3), hypothetical protein, N-acetylmannosamine-6-phosphate 2-epimerase, degenerate transposase (2) |
| 1195546-1205849 | 10.3 | 10 | hypothetical protein (5), ABC transporter ATP-binding protein, drug efflux ABC transporter, ATP-binding/permease protein, prolyl oligopeptidase family protein |
| 1268214-1270759 | 2.5 | 2 | choline binding protein |
| 1279559-1292573 | 13 | 11 | Protease, Type II restriction endonuclease (3), hypothetical protein (3), ABC transporter ATP-binding protein (3), hypothetical proteins, degenerate transposase (2) |
| 1330104-1333603 | 3.5 | 5 | cell wall surface anchor family protein, hypothetical proteins, degenerate transposase (2) |
| 1381557-1384182 | 2.4 | 1 | hypothetical protein |
| 1414003-1416052 | 2 | 1 | 1,4-beta-N-acetylmuramidase |
| 1460011-1462499 | 2.5 | 3 | hypothetical proteins |
| 1510861-1515990 | 5.1 | 5 | ABC transporter permease (2), ABC transporter substrate-binding protein, hypothetical protein, sialidase A precursor (neuraminidase A) |
| 1529462-1533414 | 4 | 5 | ABC transporter ATP-binding protein, hypothetical prot. (4) |
| 1590612-1592989 | 2.3 | 3 | sucrose-6-phosphate hydrolase, putative, ABC transporter membrane-spanning permease - sugar transport |
| 1602308-1604450 | 2.1 | 3 | transcriptional regulator, hypothetical protein |
| 1611600-1613961 | 2.3 | 3 | catabolite control protein, hypothetical protein, Mg2+ transporter |
| 1682796-1684507 | 1.7 | 1 | sugar ABC transporter, permease protein (2) |

| | | | |
|---|---|---|---|
| 1691699-1697659 | 6 | 0 | Not annotated |
| 1713561-1722800 | 9.2 | 13 | Pneumolysin, hypothetical proteins (5), degenerate transposase (orf1/2), N-acetylmuramoyl-L-alanine amidase |
| 1729774-1741445 | 11.7 | 13 | hypothetical proteins (6), transcriptional activator, bacteriocin formation protein, putative, toxin secretion ABC transporter, ATP-binding/permease protein, subtilisin-like serine protease |
| 1771172-1775517 | 4.4 | 4 | hypothetical protein (2), ABC transporter ATP-binding protein - unknown substrate, transcriptional regulator PlcR, putative |
| 1788552-1795751 | 7.2 | 0 | Not annotated<br>BlastX indicates similarity to a Competence-specific global transcription modulator |
| 1802154-1807851 | 5.7 | 7 | Transposase (2), nicotinate-nucleotide pyrophosphorylase, hypothetical proteins (3), Beta-glucosidase |
| 1808352-1811677 | 3.3 | 3 | PTS system, IIC, B and A components |
| 1851852-1856497 | 4.3 | 0 | Not annotated |
| 1871050-1877198 | 6.1 | 7 | tranposase (orf2), hypothetical protein, response regulator sensor histidine kinase PnpS, phosphate ABC transporter phosphate-binding protein (4) |
| 1877695-1879688 | 2 | | phosphate transporter PhoUm, truncated IS1380-Spn1 transposase, transcriptional regulator |
| 1893316-1895607 | 2.3 | 3 | hypothetical protein (2) |
| 1917722-1920516 | 2.8 | 4 | transketolase, C-terminal subunit, putative transketolase n-terminal section, PTS system ascorbate-specific transporter subunit IIC |
| 1920916-1923966 | 3 | 5 | PTS system, IIB component, putative, hypothetical prot. (2) |
| 1924635-1929904 | 5.3 | 6 | hypothetical protein, 50S ribosomal protein L32 and L33, choline binding protein PcpA, degenerate transposase (orf1), tranposase (orf2), hypothetical protein |
| 1953474-1965988 | 12.5 | 13 | fucolectin-related protein, hypothetical protein, PTS system, IIA, B, C and D components, fucose pathway protein, function unknown, L-fuculose phosphate aldolase, fucose kinase, fucose operon repressor, putative |
| 1974387-1978096 | 3.7 | 2 | hypothetical proteins |
| 1987348-1992052 | 4.7 | 6 | choline binding protein A, hypothetical protein, histidine kinase, response regulator |

## *Neisseria_meningitidis* FAM18 vs oral metagenome

| | | | |
|---|---|---|---|
| 46181-52657 | 6.5 | 2 | Non-annotated region (5 kb), hypothetical protein, putative DNA transport competence protein. |
| 55856-59447 | 3.6 | 5 | LPS ISLAND. Putative inner membrane transport protein, dTDP-4-dehydrorhamnose 3,5-epimerase, glucose-1-phosphate thymidylyltransferase, dTDP-glucose 4,6-dehydratase, UDP-glucose 4-epimerase. |
| 59826-68935 | 9.1 | 7 | Hypothetical protein, alpha-2,9-polysialyltransferase, N- |

| | | | |
|---|---|---|---|
| | | | acetylneuraminic acid synthetase, acylneuraminate cytidylyltransferase, N-acetylglucosamine-6-phosphate 2-epimerase, capsule polysaccharide export outer membrane and inner membrane protein (2) |
| 73146-82686 | 9.5 | 6 | capsule polysaccharide modification protein (2), dTDP-D-glucose 4,6-dehydratase, dTDP-glucose 4,6-dehydratase, glucose-1-phosphate thymidylyltransferase, hypothetical protein. |
| 89795-96284 | 6.5 | 8 | hypothetical protein (3), IS1016 transposase partial CDS, putative inner membrane protein (2), putative protein export protein (2). |
| 207663-210169 | 2.5 | 3 | hypothetical protein, putative integral membrane protein, class II pilin PilE. |
| 213814-216632 | 2.8 | 1 | RNA polymerase sigma factor solo coge 400pb, 2388pb sin hits adicionales |
| 236567-239209 | 2.6 | 2 | Putative inner membrane protein (2) |
| 278434-288427 | 10 | 12 | hypothetical protein (6), putative inner membrane protein (2), putative invertase/transposase, putative periplasmic protein, putative rotamase, TspB protein. |
| 374923-381021 | 6.1 | 2 | Hypothetical protein, pilus-associated protein. |
| 454574-485141 | 30.6 | 23 | Hemagglutination island. Hypothetical protein (14), N-acetyl-gamma-glutamyl-phosphate reductase, putative hemagglutinin (2), putative hemagglutinin/hemolysin-related protein (2), putative hemolysin activator, lipoprotein, periplasmic protein, secretion protein. |
| 551138-561195 | 10 | 4 | Putative peptidase, putative transposase, putative outer membrane protein, iron-regulated protein FrpC. |
| 620616-625974 | 5.4 | 8 | Adhesin Maf (2), hypothetical protein (5), ribonuclease inhibitor barstar. |
| 669848-675838 | 6 | 1 | IgA1 protease |
| 810425-817429 | 7 | 11 | Hypothetical protein (8), putative integral membrane protein, putative periplasmic protein (2). |
| 847060-877132 | 30 | 35 | Hypothetical protein (23), putative D-lactate dehydrogenase-related protein, putative lipoprotein, putative Phage integrase, putative phage related protein (8), transcriptional regulator. |
| 883641-891278 | 7.6 | 8 | HlyD family secretion protein, hypothetical protein (2), ParA protein, putative ABC transporter, putative integral membrane protein (2), putative ParB protein. |
| 899469-904123 | 4.5 | 3 | putative acyl-CoA hydrolase, pseudogene (opacity protein), putative transposase for IS1655. |
| 1038354-1046140 | 7.8 | 8 | Phage island.<br>Hypothetical protein (5), putative host-nuclease inhibitor protein, phage tail fibre protein, transposase for IS1655. |
| 1299450-1302814 | 3.4 | 1 | Putative type III restriction/modification system enzyme |
| 1327713-1338626 | 10.9 | 3 | putative cytolysin secretion ABC transporter, insertion element IS1016 transposase, putative RTX iron-regulated frpc protein outer membrane. |
| 1401490-1405236 | 3.7 | 2 | Pseudogene (opacity protein), hypothetical protein. |
| 1480022-1489392 | 9.4 | 2 | Lactoferrin binding protein |

| | | | |
|---|---|---|---|
| 1599194-1605603 | 6.4 | 2 | Hypothetical protein, putative DNA transport competence protein. |
| 1692689-1700188 | 4.5 | 10 | Hypothetical protein (5), putative cell-surface protein, putative integral membrane protein (2), putative periplasmic type I secretion system protein (2). |
| 1741953-1754180 | 12.2 | 11 | Hypothetical protein (5), Opa1800 outer membrane protein precursor, putative integral membrane protein (3), putative transposase, TspB protein. |
| 1833155-1844849 | 11.7 | 17 | Adhesin Maf (2), hypothetical protein (14), putative lipoprotein. |
| 1890038-1902422 | 12.4 | 10 | Hypothetical protein (5), putative integral membrane protein (2), putative invertase/transposase, putative replication initiation factor, TspB protein. |
| 1905347-1910613 | 5.3 | 5 | Hypothetical protein (3), pseudogene (outer membrane protein), putative DNA transport competence protein. |
| 1965871-1971037 | 5.2 | 1 | Putative outer membrane peptidase |
| 1990115-1996307 | 6.2 | 2 | Hypothetical protein, Ig-Aspecific serine endopeptidase. |
| 2040719-2048085 | 7.4 | 3 | Hypothetical protein, putative pilin, putative DNA transport competence protein. |
| 2133941-2143373 | 9.4 | 15 | Adhesin (2), hypothetical protein (12), mafb protein (fragment). |
