# Supplementary material for: Mining Virulence Genes Using Metagenomics
Source: PLoS One. 2011 Oct 19;6(10):e24975. doi: 10.1371/journal.pone.0024975 (PMC3198465; doi:10.1371/journal.pone.0024975)
Supplement: Table S2 — Full gene content of Metagenomic Islands detected by recruitment of selected virulence plasmids from enteric bacteria against the gut metagenome. (PDF) [file pone.0024975.s003.pdf]

Supplementary Table 2. Full gene content of Metagenomic Islands detected by recruitment of selected virulence plasmids from enteric bacteria against the gut metagenome.

PLASMIDS	Start-End (bp)	Length (kbp)	Number of ORFs	Main features (number of genes)
Escherichia coli E24377A plasmid pETEC_35	7893-11662	3.8	1	Conjugal transfer mating pair stabilization protein TraG.
	20410-25193	1.7	4	Protein stbB, plasmid segregation protein ParM, hypothetical protein (2).
	26190-28154	2	2	Hypothetical protein (2)
	30914-31839	0.9	1	Hypothetical protein
	32009-33237	1.2	1	Type-F conjugative transfer system pilin acetylase TraX
Escherichia coli E24377A plasmid pETEC_73	8008-11232	3.2	2	Stable plasmid inheritance protein, plasmid segregation protein ParM.
	13750-16212	2.5	1	Hypothetical protein
	20504-24114	3.6	3	Hypothetical protein, AraC family transcriptional regulator, IS186 transposase.
	25124-26985	1.9	1	AraC family transcriptional regulator
	30107-36352	6.2	4	Fimbrial protein, fimbrial usher protein, hypothetical protein (2).
	38174-38683	0.5	1	Hypothetical protein
	40877-41739	0.8	3 (overlapping)	Hypothetical protein (3)
	42663-43652	0.9	1	Replication initiation protein
	44305-45616	1.3	0	Not annotated
	46134-50395	4.3	3	Hypothetical protein, PilL protein (outer membrane lipoprotein), PilM protein (inner membrane protein).<br>(Components of the pilin transport apparatus and thin-pilus basal body)
Escherichia coli E24377A plasmid pETEC_74	1838-2595	0.8	1	IS1294 transposase truncation
	3101-6084	3	3	IS66 family orf1, 2 and transposase
	6825-8563	1.7	3	IS91 transposase truncation (2), IS91 orf2.
	15635-18183	2.5	2	IS21 family transposition helper protein, IS21 family transposase.
	26267-27233	1	0	Nothing anotated
	36654-41560	4.9	4	Hypothetical protein, IS66 family transposase, IS66 family orf 1 and 2
	43942-46420	2.5	3	IS66 family transposase, IS66 family orf 1 & 2
	47949-51799	2.9	1 (parcial)	Secreted serine peptidase EatA
	54192-55795	1.6	1	EAL domain-containing protein
	66782-69352	2.6	7	IS66 family orf2, IS66 family element, hypothetical protein (2), type IV conjugative transfer system pilin TraA, conjugal transfer pilus assembly protein TraL, conjugal transfer pilus assembly protein TraE
Escherichia coli E24377A plasmid pETEC_80	0-4061	4	3	Hypothetical protein (3)
	6954-9769	2.8	2	Plasmid segregation protein ParM, hypothetical protein.
	10564-11701	1.1	1	IS3 family transposase
	16314-18529	2.2	2	ISSd1 transposase, heat-labile enterotoxin A
	28259-29461	1.2	3	Hypothetical protein (3)

Supplementary Table 2. Full gene content of Metagenomic Islands detected by recruitment of selected virulence plasmids from enteric bacteria against the gut metagenome.

	42046-43642	1.6	2	ParA protein, putative 60kDa chaperonin
	48647-53360	4.7	1	Periplasmic pilus chaperone CS3-1
	53966-55474	1.5	1	IS91 orf2
	56403-59240	2.8	2	IS91 orf2, AraC family transcription regulator
	60102-63660	3.6	3	Putative efflux ABC transporter permease protein, hypothetical protein (2).
	69973-71318	1.3	1	Hypothetical protein
	72739-74305	1.6	2	IS3 transposase orfA, IS91 orf2
	74977-79237	4.2	1	Hypothetical protein.
Shigella flexneri 2a plasmid pCP301	937-3098	2.1	2	OspB protein secreted by the Mxi-Spa secretion machinery of unknown function, PhoN2 (Apy) periplasmic phosphatase apyrase ATP diphosphohydrolase.
	3345-5289	1.9	1	OspC4
	5827-6942	1.1	2	IS600 Orf2 (2)
	7331-10381	3	2	Hypothetical protein (ospD2), OspF.
	10976-12301	1.3	2	Putative IS1 orf, hypothetical protein.
	17214-21768	4.5	4	Putative transposase, hypothetical protein (2), OspD1, secreted by the Mxi-Spa secretion machinery, function unknown.
	28145-29840	1.7	3	Plasmid segregation protein, hypothetical protein, putative transposase
	33662-35319	1.7	3	IS150 orfb, IS1294 transposase (2).
	36119-37666	1.5	2	Is100 ORF2, transcriptional activator VirF.
	39418-42882	3.5	1	Invasion plasmid antigen.
	44241-49514	5.3	8	IS600 ORF (2), hypothetical protein (4), IS1294 transposase, ISSfl1 ORF2.
	56068-59597	3.5	6	Extracellular serine protease SepA, IS1294 transposase (2), IS630 orf, ISSfl1 ORF (2).
	61287-66054	4.8	3	Iso-IS1 ORF2, invasion plasmid antigen (2).
	66641-71548	4.9	5	IS629ORF2, IS100 ORF1, invasion plasmid product, hypothetical protein, iso-IS10R ORF.
	72326-74542	3.2	3	IS629 ORF (2), transposase/IS protein.
	75019-81884	6.9	4	Iso-IS10R ORF, IS1294 transposase, hypothetical protein, OspC1 secreted by the Mxi-Spa secretion machinery, function unknown.
	82165-84136	2	5	Hypothetical protein (2), putative IS91 ORF2, ISSfl1 ORF (2),
	90839-98072	7.2	7	Hypothetical protein, IS600 ORF (2), ISSfl4 ORF (3), OspC3.
	102451-126782	24.3	25	Acp, putative acyl carrier protein, AraC-like family protein MxiE, hypothetical protein (3), IcsB invasion protein, IpaB & IpaC secreted by the Mxi-Spa secretion machinery required for entry into epithelial cells, IpaJ invasion plasmid antigen, IpgA, IpgB1 secreted by the Mxi-Spa machinery function unknown, IpgC cytoplasmic chaperone for IpaB and IpaC, IpgE cytoplasmic chaperone for IpgD, IpgF periplasmic protein similarities to the catalytic site of lyzozymes, Mxi-Spa secretion

Supplementary Table 2. Full gene content of Metagenomic Islands detected by recruitment of selected virulence plasmids from enteric bacteria against the gut metagenome.

				machinery protein, MxiG, MxiH component of the Mxi-Spa secretion machinery, MxiI component of the Mxi-Spa secretion machinery, MxiJ lipoprotein component of the Mxi-Spa secretion machinery, MxiK putative component of the Mxi-Spa secretion machinery, MxiL/M/N secreted by and putative component of the Mxi-Spa secretion machinery, outermembrane protein MxiD transcriptional activator VirB.
	129578-139352	9.8	14	ATP synthase SpaL, hypothetical protein, IS1294 transposase (2), IS600 ORF (3), Spa components of the Mxi-Spa secretion machinery (5), surface presentation of antigens protein Spa (2).
	139926-144074	4.1	6	Hypothetical protein (4), IS629 ORF (2).
	144514-146272	1.8	2	IS91 ORF (2).
	147810-153530	5.7	2	Hypothetical protein, VirA secreted by the Mxi-Spa secretion machinery function unknown.
	159998-163019	3	5	ISSfl1 ORF (2), hypothetical protein, plasmid stable inheritance protein (2).
	167751-170456	2.7	3	ISSfl4 ORF (3)
	175894-177367	1.5	3	Hypothetical protein (2), IS100 ORF.
	181073-183119	2	1	Invasion plasmid antigen secreted by the Mxi-Spa secretion machinery.
	188292-191410	3.1	2	IS3 ORF, hypothetical protein.
	195630-197563	1.9	1	IS630 orf
	211736-216287	4.6	5	Hypothetical protein, invasion plasmid antigen secreted by the Mxi-Spa secretion machinery, IS1294 transposase, OspE1, putative transposase.
Escherichia coli O157H7 Sakai str. Plasmid	15764-16630	0.8	1	Hemolysin C
	17513-18193	0.7	½	Hemolysin A
	18984-19522	0.5	½	Hemolysin A
	22987-24001	1	2	Hemolysin D PapX protein
	26744-28243	1.7	2	Hypothetical protein, KfrAs
	34595-35315	0.7	0	
	35665-36025	0.4	0	
	53881-54873	1	1	Hypothetical protein
	55930-65830	9.9	2	Toxin B, transposase Tra5.
	74298-74954	0.7	2	Hypothetical protein (2)
	79088-79552	0.5	1	Hypothetical protein
	81698-82863	1.2	1	Serine protease EspP
	85474-86980	1.5	0	
	92149-2919	0.6	1	ToxR-regulated lipoprotein
