# Supplementary material for: Mining Virulence Genes Using Metagenomics
Source: PLoS One. 2011 Oct 19;6(10):e24975. doi: 10.1371/journal.pone.0024975 (PMC3198465; doi:10.1371/journal.pone.0024975)
Supplement: Table S3 — List of strains analyzed in the manuscript and their corresponding NCBI accession numbers. (PDF) [file pone.0024975.s004.pdf]

Supplementary Table 3. List of strains analyzed and their corresponding accession numbers.

STRAIN	GENOMIC ELEMENT	ACCESSION NUMBER
Shigella flexneri 2a str 301	Chromosome	NC_004337.1
	Plasmid pCP301	NC_004851.1
Escherichia coli E243777A ETEC	Chromosome	NC_009801.1
	Plasmid pETEC_80	NC_009786.1
	Plasmid pETEC_74	NC_009790.1
	Plasmid pETEC_73	NC_009788.1
	Plasmid pETEC_35	NC_009787.1
Escherichia coli CFT073	Chromosome	NC_004431.1
Escherichia coli K12 MG1655	Chromosome	NC_000913.2
Escherichia coli O127:H6 str. E2348/69	Plasmid pMAR2	NC_011603.1
Escherichia coli O157:H7 Sakai str.	Chromosome	BA000007.2
Salmonella enterica subsp. enterica serovar Typhi str. CT18	Chromosome	NC_003198.1
Neisseria meningitidis 053442	Chromosome	NC_010120.1
Neisseria meningitidis FAM18	Chromosome	NC_008767.1
Neisseria gonorrhoeae FA1090	Chromosome	NC_002946.2
Streptococcus sanguinis SK36	Chromosome	NC_009009.1
Streptococcus pneumoniae R6	Chromosome	NC_003098.1
